# Ionic mechanisms of action potential propagation velocity changes in peripheral C-fibers. Implications for pain

**DOI:** 10.1186/1471-2202-13-S1-P138

**Published:** 2012-07-16

**Authors:** Sten A Andersson, Marcus E Petersson, Erik Fransén

**Affiliations:** 1 Department of Computational Biology, School of Computer Science and Communication, KTH Royal Institute of Technology, Stockholm, Sweden

## 

C-fibers, nonmyelinated afferent axons, convey information from the periphery of the nervous system to the spinal cord. They transmit signals originating from stimulus of temperature, pressure and tissue damage producing sensations of itch and pain. In pain, it is generally believed that underlying mechanisms include changes in ion channel conductances. These changes should be reflected in changes in the membrane potential. However, the small diameter of the fibers makes experimental measurements of the intraaxonal membrane potential intractable. Measurable properties are the conduction velocity (CV) and the activity depending slowing of velocity (ADS). ADS is changed in pain. ADS is known to be dependent on CV [4] which in turn has been suggested to be dependent on the conductances of the voltage gated sodium channels[1][2][3]. The objective of this study is to elucidate which sodium channel subtype that is the most contributing to CV and also the subtypes respective role in ADS.

In our study we represent the axon by three cylindrical sections, one representing the periperal thin end (branch, 2.5 cm), one the central part (parent, 1 cm) and a conical section between these (0.5 cm). In total 730 compartments are used. We represent variable ion concentrations of Na and K intra axonally, periaxonally and extracellularly, from which reversal potentials are calculated. An ion pump (Na/K-ATPase) is included. Temperature is set to 32 degrees C in branch and 37 degrees in parent sections. We use ion channel models based on Hodgkin Huxley formalism. We use three voltage gated sodium channels (TTX-sensitive Na, Na_v_1.8, Na_v_1.9), four voltage gated potassium channels (K_A_, K_M_, delayed rectifier K_dr_, sodium dependent K), as well as h and leak channels. Conduction velocity is measured by eliciting an action potential in the peripheral end of the axon and measuring the time delay to a point near the spinal cord. In the first step, we compare our detailed model to macroscopic relations for CV derived in classical cable theory. We study the following parameters: membrane capacitance (C_m_), axial resistance (R_a_), axon diameter (D) and membrane resistance (R_m_). The second step analyzes which ion channel subtypes which give the maximum contribution to CV. We further study ion channel contributions to ADS.

## Conclusions

We find that TTX- sensitive Na and Na_v_1.8 have the strongest influence on CV as is expected since these are the major components of the rising phase of the action potential. This is also consistent with proposals for more simplified models [1][2][3]. For a set of TTX-sensitive Na conductances values we find that CV has negative correlation to ADS and from a set of Nav1.8 conductances we find that CV has positive correlation to ADS. Our findings demonstrate that detailed conductance based modeling can provide insights of the causal relationship between CV and ADS and the underlying ion channel conductances. In forthcoming studies, changes in ion channels observed in pain patients will be compared to corresponding model outcomes.
